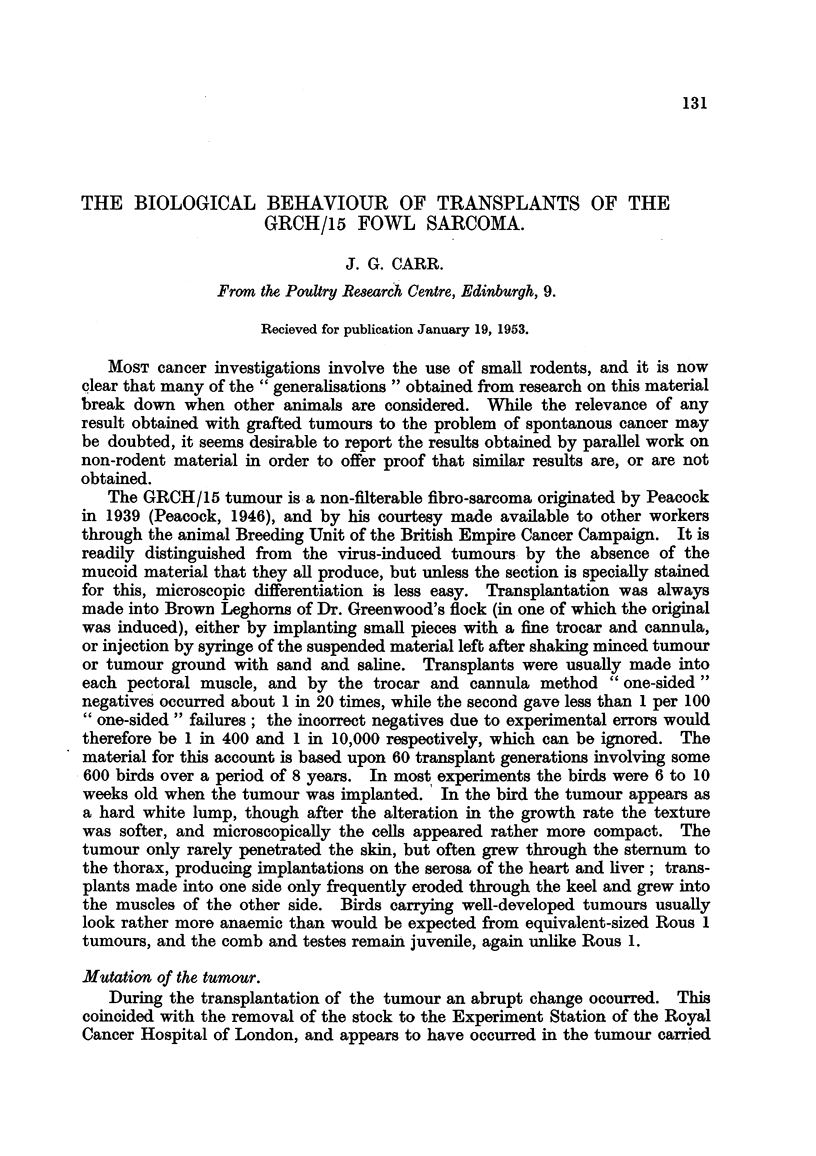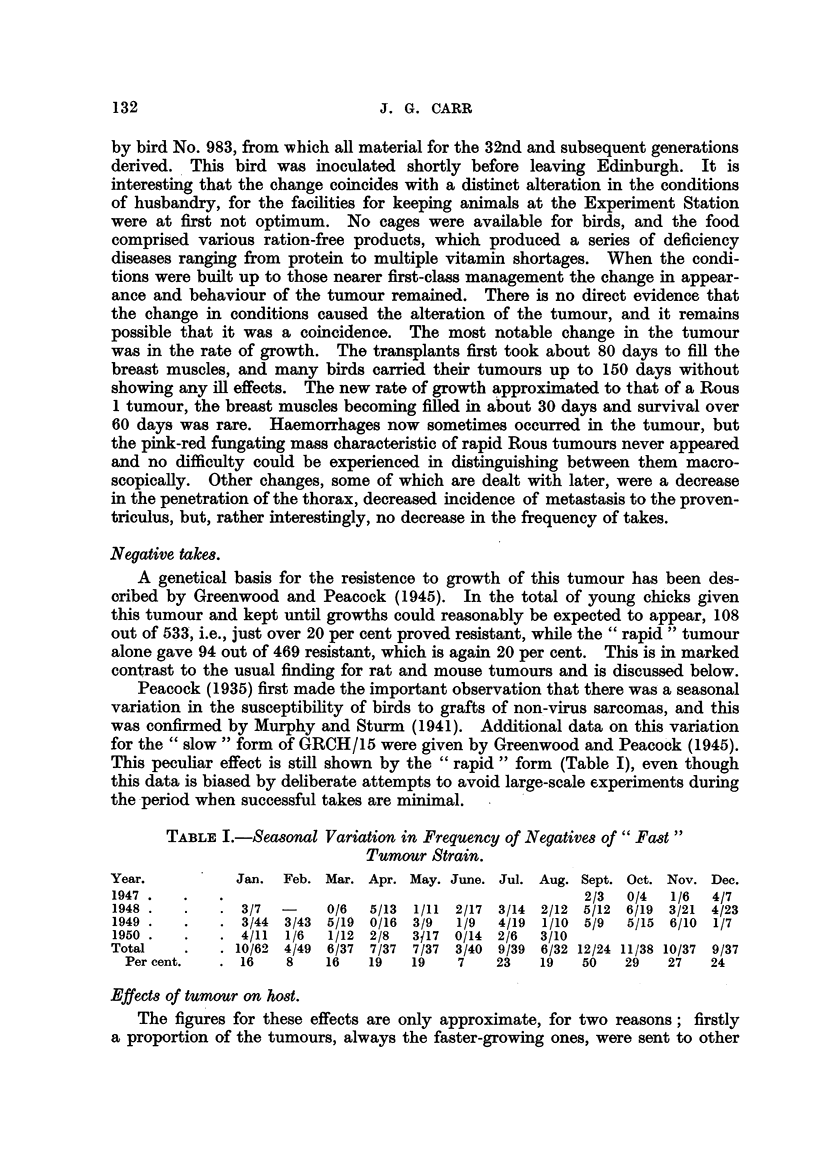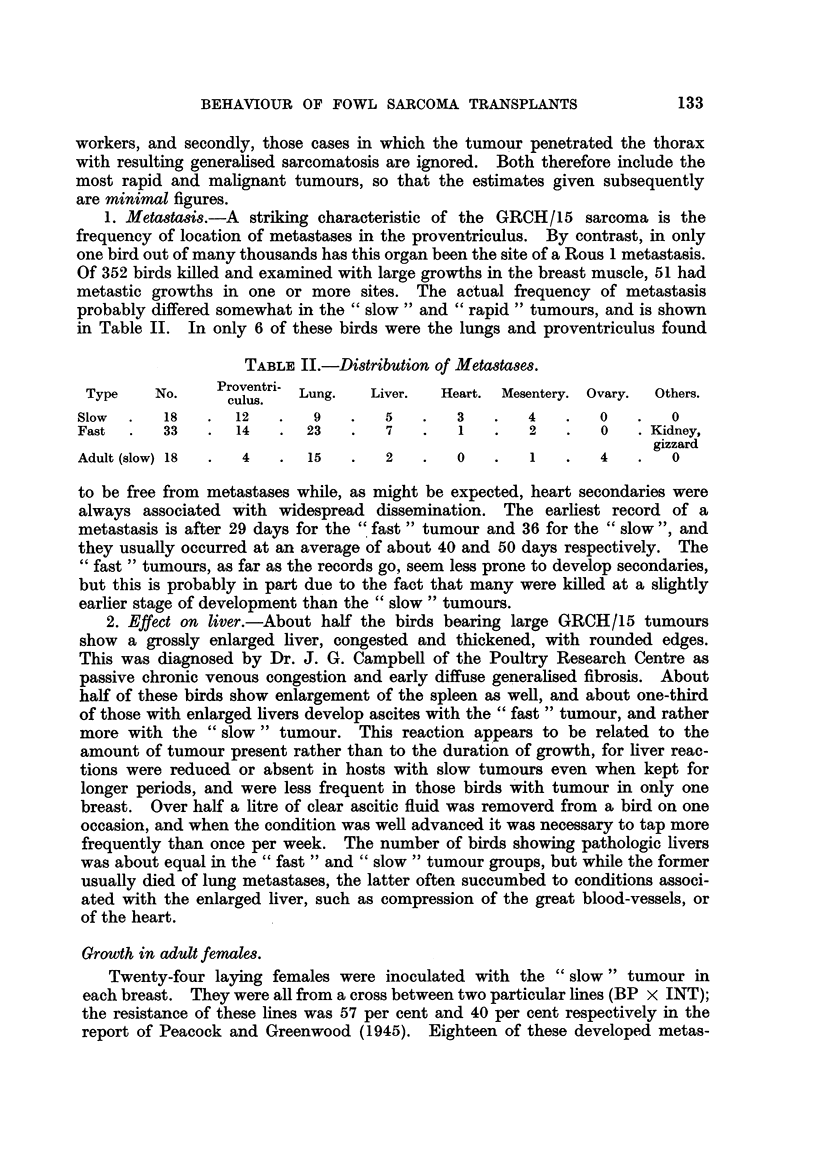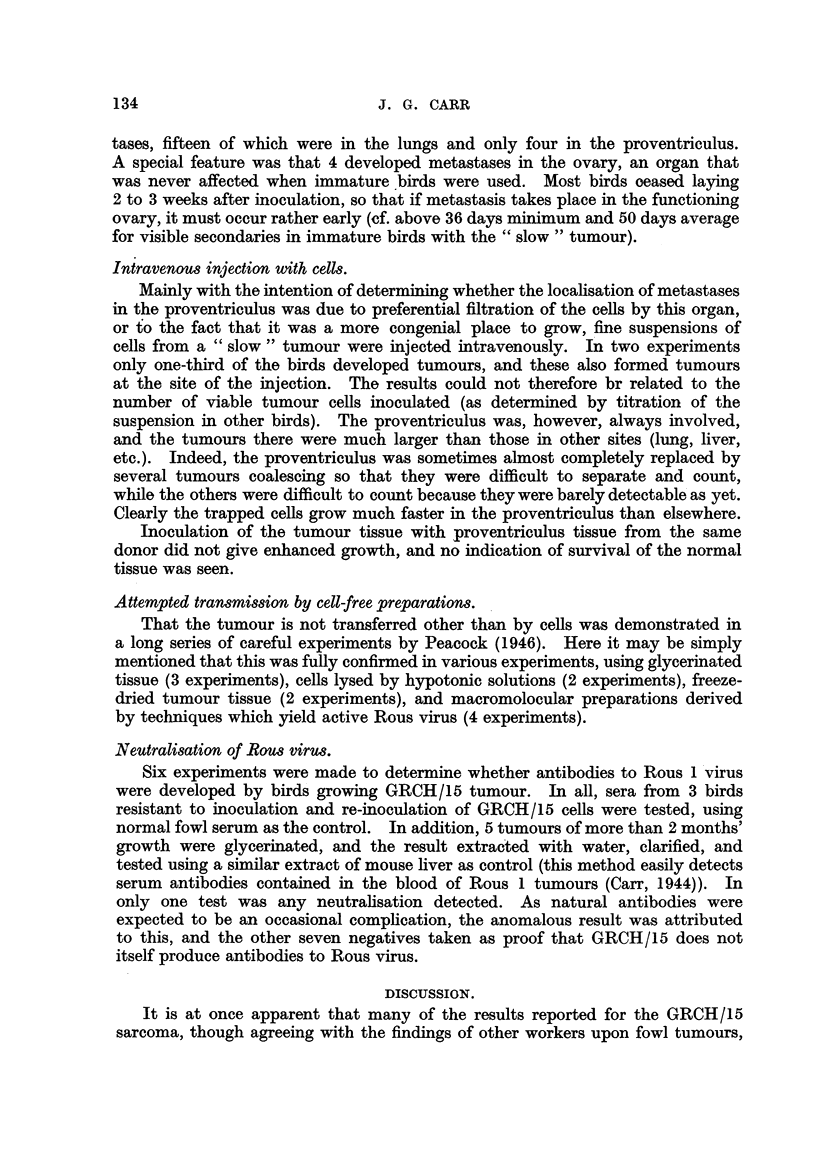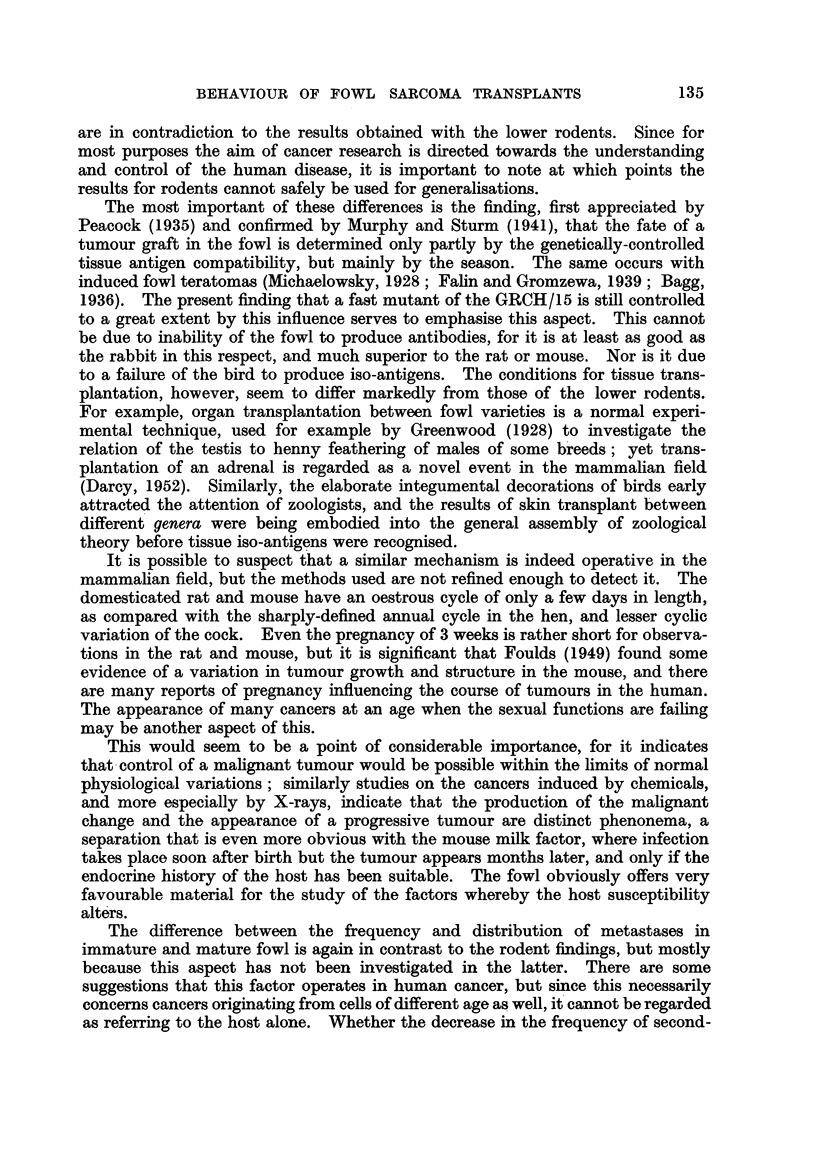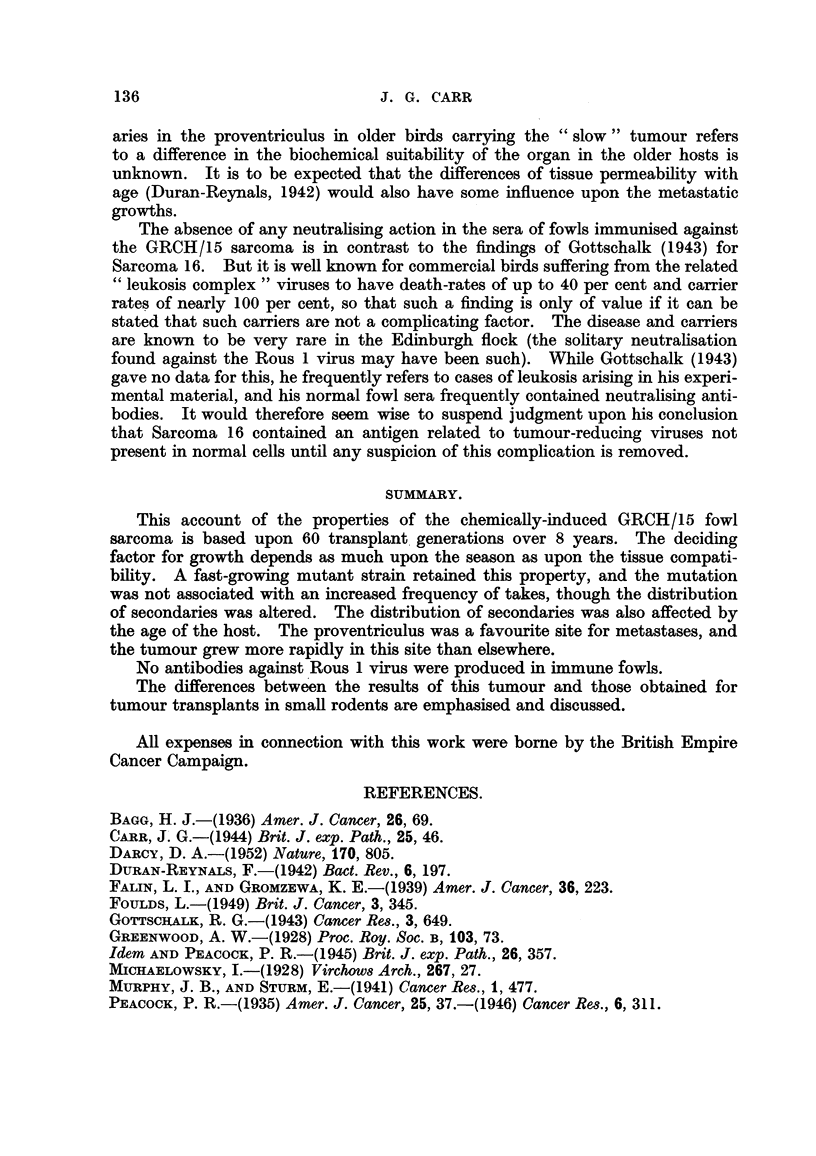# The Biological Behaviour of Transplants of the GRCH/15 Fowl Sarcoma

**DOI:** 10.1038/bjc.1953.11

**Published:** 1953-03

**Authors:** J. G. Carr


### THE BIOLOGICAL BEHAVIOUR OF TRANSPLANTS OF THE GRCH/15 FOWL SARCOMA.

J. G. CARR.

*From the Poultry Research Centre, Edinburgh, 9.*

Recieved for publication January 19, 1953.

MOST cancer investigations involve the use of small rodents, and it is now clear that many of the " generalisations " obtained from research on this material break down when other animals are considered. While the relevance of any result obtained with grafted tumours to the problem of spontanous cancer may be doubted, it seems desirable to report the results obtained by parallel work on non-rodent material in order to offer proof that similar results are, or are not obtained.

The GRCH/15 tumour is a non-filterable fibro-sarcoma originated by Peacock in 1939 (Peacock, 1946), and by his courtesy made available to other workers through the animal Breeding Unit of the British Empire Cancer Campaign. It is readily distinguished from the virus-induced tumours by the absence of the mucoid material that they all produce, but unless the section is specially stained for this, microscopic differentiation is less easy. Transplantation was always made into Brown Leghorns of Dr. Greenwood's flock (in one of which the original was induced), either by implanting small pieces with a fine trocar and cannula, or injection by syringe of the suspended material left after shaking minced tumour or tumour ground with sand and saline. Transplants were usually made into each pectoral muscle, and by the trocar and cannula method " one-sided " negatives occurred about 1 in 20 times, while the second gave less than 1 per 100 " one-sided " failures ; the incorrect negatives due to experimental errors would therefore be 1 in 400 and 1 in 10,000 respectively, which can be ignored. The material for this account is based upon 60 transplant generations involving some 600 birds over a period of 8 years. In most experiments the birds were 6 to 10 weeks old when the tumour was implanted. In the bird the tumour appears as a hard white lump, though after the alteration in the growth rate the texture was softer, and microscopically the cells appeared rather more compact. The tumour only rarely penetrated the skin, but often grew through the sternum to the thorax, producing implantations on the serosa of the heart and liver ; transplants made into one side only frequently eroded through the keel and grew into the muscles of the other side. Birds carrying well-developed tumours usually look rather more anaemic than would be expected from equivalent-sized Rous 1 tumours, and the comb and testes remain juvenile, again unlike Rous 1.

*Mutation of the tumour.*

During the transplantation of the tumour an abrupt change occurred. This coincided with the removal of the stock to the Experiment Station of the Royal Cancer Hospital of London, and appears to have occurred in the tumour carried by bird No. 983, from which all material for the 32nd and subsequent generations derived. This bird was inoculated shortly before leaving Edinburgh. It is interesting that the change coincides with a distinct alteration in the conditions of husbandry, for the facilities for keeping animals at the Experiment Station were at first not optimum. No cages were available for birds, and the food comprised various ration-free products, which produced a series of deficiency diseases ranging from protein to multiple vitamin shortages. When the conditions were built up to those nearer first-class management the change in appearance and behaviour of the tumour remained. There is no direct evidence that the change in conditions caused the alteration of the tumour, and it remains possible that it was a coincidence. The most notable change in the tumour was in the rate of growth. The transplants first took about 80 days to fill the breast muscles, and many birds carried their tumours up to 150 days without showing any ill effects. The new rate of growth approximated to that of a Rous 1 tumour, the breast muscles becoming filled in about 30 days and survival over 60 days was rare. Haemorrhages now sometimes occurred in the tumour, but the pink-red fungating mass characteristic of rapid Rous tumours never appeared and no difficulty could be experienced in distinguishing between them macroscopically. Other changes, some of which are dealt with later, were a decrease in the penetration of the thorax, decreased incidence of metastasis to the proventriculus, but, rather interestingly, no decrease in the frequency of takes.

*Negative takes.*

A genetical basis for the resistence to growth of this tumour has been described by Greenwood and Peacock (1945). In the total of young chicks given this tumour and kept until growths could reasonably be expected to appear, 108 out of 533, i.e., just over 20 per cent proved resistant, while the " rapid " tumour alone gave 94 out of 469 resistant, which is again 20 per cent. This is in marked contrast to the usual finding for rat and mouse tumours and is discussed below.

Peacock (1935) first made the important observation that there was a seasonal variation in the susceptibility of birds to grafts of non-virus sarcomas, and this was confirmed by Murphy and Sturm (1941). Additional data on this variation for the " slow " form of GRCH/15 were given by Greenwood and Peacock (1945). This peculiar effect is still shown by the " rapid " form (Table I), even though this data is biased by deliberate attempts to avoid large-scale experiments during the period when successful takes are minimal.

TABLE I.—*Seasonal Variation in Frequency of Negatives of " Fast " Tumour Strain.*

| Year. | Jan. | Feb. | Mar. | Apr. | May. | June. | Jul. | Aug. | Sept. | Oct. | Nov. | Dec. |
|---|---|---|---|---|---|---|---|---|---|---|---|---|
| 1947 . . . | | | | | | | | | 2/3 | 0/4 | 1/6 | 4/7 |
| 1948 . . . | 3/7 | — | 0/6 | 5/13 | 1/11 | 2/17 | 3/14 | 2/12 | 5/12 | 6/19 | 3/21 | 4/23 |
| 1949 . . . | 3/44 | 3/43 | 5/19 | 0/16 | 3/9 | 1/9 | 4/19 | 1/10 | 5/9 | 5/15 | 6/10 | 1/7 |
| 1950 . . . | 4/11 | 1/6 | 1/12 | 2/8 | 3/17 | 0/14 | 2/6 | 3/10 | | | | |
| Total . . | 10/62 | 4/49 | 6/37 | 7/37 | 7/37 | 3/40 | 9/39 | 6/32 | 12/24 | 11/38 | 10/37 | 9/37 |
| Per cent. . | 16 | 8 | 16 | 19 | 19 | 7 | 23 | 19 | 50 | 29 | 27 | 24 |

*Effects of tumour on host.*

The figures for these effects are only approximate, for two reasons ; firstly a proportion of the tumours, always the faster-growing ones, were sent to other workers, and secondly, those cases in which the tumour penetrated the thorax with resulting generalised sarcomatosis are ignored. Both therefore include the most rapid and malignant tumours, so that the estimates given subsequently are *minimal* figures.

1. *Metastasis.*—A striking characteristic of the GRCH/15 sarcoma is the frequency of location of metastases in the proventriculus. By contrast, in only one bird out of many thousands has this organ been the site of a Rous 1 metastasis. Of 352 birds killed and examined with large growths in the breast muscle, 51 had metastic growths in one or more sites. The actual frequency of metastasis probably differed somewhat in the " slow " and " rapid " tumours, and is shown in Table II. In only 6 of these birds were the lungs and proventriculus found

TABLE II.—*Distribution of Metastases.*

| Type | No. | Proventriculus. | Lung. | Liver. | Heart. | Mesentery. | Ovary. | Others. |
|---|---|---|---|---|---|---|---|---|
| Slow | 18 | 12 | 9 | 5 | 3 | 4 | 0 | 0 |
| Fast | 33 | 14 | 23 | 7 | 1 | 2 | 0 | Kidney, gizzard |
| Adult (slow) | 18 | 4 | 15 | 2 | 0 | 1 | 4 | 0 |

to be free from metastases while, as might be expected, heart secondaries were always associated with widespread dissemination. The earliest record of a metastasis is after 29 days for the " fast " tumour and 36 for the " slow ", and they usually occurred at an average of about 40 and 50 days respectively. The " fast " tumours, as far as the records go, seem less prone to develop secondaries, but this is probably in part due to the fact that many were killed at a slightly earlier stage of development than the " slow " tumours.

2. *Effect on liver.*—About half the birds bearing large GRCH/15 tumours show a grossly enlarged liver, congested and thickened, with rounded edges. This was diagnosed by Dr. J. G. Campbell of the Poultry Research Centre as passive chronic venous congestion and early diffuse generalised fibrosis. About half of these birds show enlargement of the spleen as well, and about one-third of those with enlarged livers develop ascites with the " fast " tumour, and rather more with the " slow " tumour. This reaction appears to be related to the amount of tumour present rather than to the duration of growth, for liver reactions were reduced or absent in hosts with slow tumours even when kept for longer periods, and were less frequent in those birds with tumour in only one breast. Over half a litre of clear ascitic fluid was removerd from a bird on one occasion, and when the condition was well advanced it was necessary to tap more frequently than once per week. The number of birds showing pathologic livers was about equal in the " fast " and " slow " tumour groups, but while the former usually died of lung metastases, the latter often succumbed to conditions associated with the enlarged liver, such as compression of the great blood-vessels, or of the heart.

*Growth in adult females.*

Twenty-four laying females were inoculated with the " slow " tumour in each breast. They were all from a cross between two particular lines (BP × INT); the resistance of these lines was 57 per cent and 40 per cent respectively in the report of Peacock and Greenwood (1945). Eighteen of these developed metastases, fifteen of which were in the lungs and only four in the proventriculus. A special feature was that 4 developed metastases in the ovary, an organ that was never affected when immature birds were used. Most birds ceased laying 2 to 3 weeks after inoculation, so that if metastasis takes place in the functioning ovary, it must occur rather early (cf. above 36 days minimum and 50 days average for visible secondaries in immature birds with the " slow " tumour).

*Intravenous injection with cells.*

Mainly with the intention of determining whether the localisation of metastases in the proventriculus was due to preferential filtration of the cells by this organ, or to the fact that it was a more congenial place to grow, fine suspensions of cells from a " slow " tumour were injected intravenously. In two experiments only one-third of the birds developed tumours, and these also formed tumours at the site of the injection. The results could not therefore br related to the number of viable tumour cells inoculated (as determined by titration of the suspension in other birds). The proventriculus was, however, always involved, and the tumours there were much larger than those in other sites (lung, liver, etc.). Indeed, the proventriculus was sometimes almost completely replaced by several tumours coalescing so that they were difficult to separate and count, while the others were difficult to count because they were barely detectable as yet. Clearly the trapped cells grow much faster in the proventriculus than elsewhere.

Inoculation of the tumour tissue with proventriculus tissue from the same donor did not give enhanced growth, and no indication of survival of the normal tissue was seen.

*Attempted transmission by cell-free preparations.*

That the tumour is not transferred other than by cells was demonstrated in a long series of careful experiments by Peacock (1946). Here it may be simply mentioned that this was fully confirmed in various experiments, using glycerinated tissue (3 experiments), cells lysed by hypotonic solutions (2 experiments), freeze-dried tumour tissue (2 experiments), and macromolocular preparations derived by techniques which yield active Rous virus (4 experiments).

*Neutralisation of Rous virus.*

Six experiments were made to determine whether antibodies to Rous 1 virus were developed by birds growing GRCH/15 tumour. In all, sera from 3 birds resistant to inoculation and re-inoculation of GRCH/15 cells were tested, using normal fowl serum as the control. In addition, 5 tumours of more than 2 months' growth were glycerinated, and the result extracted with water, clarified, and tested using a similar extract of mouse liver as control (this method easily detects serum antibodies contained in the blood of Rous 1 tumours (Carr, 1944)). In only one test was any neutralisation detected. As natural antibodies were expected to be an occasional complication, the anomalous result was attributed to this, and the other seven negatives taken as proof that GRCH/15 does not itself produce antibodies to Rous virus.

### DISCUSSION.

It is at once apparent that many of the results reported for the GRCH/15 sarcoma, though agreeing with the findings of other workers upon fowl tumours, are in contradiction to the results obtained with the lower rodents. Since for most purposes the aim of cancer research is directed towards the understanding and control of the human disease, it is important to note at which points the results for rodents cannot safely be used for generalisations.

The most important of these differences is the finding, first appreciated by Peacock (1935) and confirmed by Murphy and Sturm (1941), that the fate of a tumour graft in the fowl is determined only partly by the genetically-controlled tissue antigen compatibility, but mainly by the season. The same occurs with induced fowl teratomas (Michaelowsky, 1928 ; Falin and Gromzewa, 1939 ; Bagg, 1936). The present finding that a fast mutant of the GRCH/15 is still controlled to a great extent by this influence serves to emphasise this aspect. This cannot be due to inability of the fowl to produce antibodies, for it is at least as good as the rabbit in this respect, and much superior to the rat or mouse. Nor is it due to a failure of the bird to produce iso-antigens. The conditions for tissue transplantation, however, seem to differ markedly from those of the lower rodents. For example, organ transplantation between fowl varieties is a normal experimental technique, used for example by Greenwood (1928) to investigate the relation of the testis to henny feathering of males of some breeds ; yet transplantation of an adrenal is regarded as a novel event in the mammalian field (Darcy, 1952). Similarly, the elaborate integumental decorations of birds early attracted the attention of zoologists, and the results of skin transplant between different *genera* were being embodied into the general assembly of zoological theory before tissue iso-antigens were recognised.

It is possible to suspect that a similar mechanism is indeed operative in the mammalian field, but the methods used are not refined enough to detect it. The domesticated rat and mouse have an oestrous cycle of only a few days in length, as compared with the sharply-defined annual cycle in the hen, and lesser cyclic variation of the cock. Even the pregnancy of 3 weeks is rather short for observations in the rat and mouse, but it is significant that Foulds (1949) found some evidence of a variation in tumour growth and structure in the mouse, and there are many reports of pregnancy influencing the course of tumours in the human. The appearance of many cancers at an age when the sexual functions are failing may be another aspect of this.

This would seem to be a point of considerable importance, for it indicates that control of a malignant tumour would be possible within the limits of normal physiological variations ; similarly studies on the cancers induced by chemicals, and more especially by X-rays, indicate that the production of the malignant change and the appearance of a progressive tumour are distinct phenonema, a separation that is even more obvious with the mouse milk factor, where infection takes place soon after birth but the tumour appears months later, and only if the endocrine history of the host has been suitable. The fowl obviously offers very favourable material for the study of the factors whereby the host susceptibility alters.

The difference between the frequency and distribution of metastases in immature and mature fowl is again in contrast to the rodent findings, but mostly because this aspect has not been investigated in the latter. There are some suggestions that this factor operates in human cancer, but since this necessarily concerns cancers originating from cells of different age as well, it cannot be regarded as referring to the host alone. Whether the decrease in the frequency of secondaries in the proventriculus in older birds carrying the " slow " tumour refers to a difference in the biochemical suitability of the organ in the older hosts is unknown. It is to be expected that the differences of tissue permeability with age (Duran-Reynals, 1942) would also have some influence upon the metastatic growths.

The absence of any neutralising action in the sera of fowls immunised against the GRCH/15 sarcoma is in contrast to the findings of Gottschalk (1943) for Sarcoma 16. But it is well known for commercial birds suffering from the related " leukosis complex " viruses to have death-rates of up to 40 per cent and carrier rates of nearly 100 per cent, so that such a finding is only of value if it can be stated that such carriers are not a complicating factor. The disease and carriers are known to be very rare in the Edinburgh flock (the solitary neutralisation found against the Rous 1 virus may have been such). While Gottschalk (1943) gave no data for this, he frequently refers to cases of leukosis arising in his experimental material, and his normal fowl sera frequently contained neutralising antibodies. It would therefore seem wise to suspend judgment upon his conclusion that Sarcoma 16 contained an antigen related to tumour-reducing viruses not present in normal cells until any suspicion of this complication is removed.

### SUMMARY.

This account of the properties of the chemically-induced GRCH/15 fowl sarcoma is based upon 60 transplant generations over 8 years. The deciding factor for growth depends as much upon the season as upon the tissue compatibility. A fast-growing mutant strain retained this property, and the mutation was not associated with an increased frequency of takes, though the distribution of secondaries was altered. The distribution of secondaries was also affected by the age of the host. The proventriculus was a favourite site for metastases, and the tumour grew more rapidly in this site than elsewhere.

No antibodies against Rous 1 virus were produced in immune fowls.

The differences between the results of this tumour and those obtained for tumour transplants in small rodents are emphasised and discussed.

All expenses in connection with this work were borne by the British Empire Cancer Campaign.

### REFERENCES.

Bagg, H. J.—(1936) *Amer. J. Cancer*, **26**, 69.
Carr, J. G.—(1944) *Brit. J. exp. Path.*, **25**, 46.
Darcy, D. A.—(1952) *Nature*, **170**, 805.
Duran-Reynals, F.—(1942) *Bact. Rev.*, **6**, 197.
Falin, L. I., and Gromzewa, K. E.—(1939) *Amer. J. Cancer*, **36**, 223.
Foulds, L.—(1949) *Brit. J. Cancer*, **3**, 345.
Gottschalk, R. G.—(1943) *Cancer Res.*, **3**, 649.
Greenwood, A. W.—(1928) *Proc. Roy. Soc.* b, **103**, 73.
*Idem* and Peacock, P. R.—(1945) *Brit. J. exp. Path.*, **26**, 357.
Michaelowsky, I.—(1928) *Virchows Arch.*, **267**, 27.
Murphy, J. B., and Sturm, E.—(1941) *Cancer Res.*, **1**, 477.
Peacock, P. R.—(1935) *Amer. J. Cancer*, **25**, 37.—(1946) *Cancer Res.*, **6**, 311.